# Sleep Improves Memory: The Effect of Sleep on Long Term Memory in Early Adolescence

**DOI:** 10.1371/journal.pone.0042191

**Published:** 2012-08-07

**Authors:** Katya Trudeau Potkin, William E. Bunney

**Affiliations:** 1 Department of Human Biology, Brown University, Providence, Rhode Island, United States of America; 2 Department of Psychiatry and Human Behavior, University of California Irvine, Irvine, California, United States of America; University of Granada, Spain

## Abstract

Sleep plays an important role in the consolidation of memory. This has been most clearly shown in adults for procedural memory (i.e. skills and procedures) and declarative memory (e.g. recall of facts). The effects of sleep and memory are relatively unstudied in adolescents. Declarative memory is important in school performance and consequent social functioning in adolescents. This is the first study to specifically examine the effects of normal sleep on auditory declarative memory in an early adolescent sample. Given that the majority of adolescents do not obtain the recommended amount of sleep, it is critical to study the cognitive effects of normal sleep. Forty male and female normal, healthy adolescents between the ages of ten and fourteen years old were randomly assigned to sleep and no sleep conditions. Subjects were trained on a paired-associate declarative memory task and a control working memory task at 9am, and tested at night (12 hours later) without sleep. The same number of subjects was trained at 9pm and tested 9am following sleep. An increase of 20.6% in declarative memory, as measured by the number correct in a paired-associate test, following sleep was observed compared to the group which was tested at the same time interval without sleep (p<0.03). The performance on the control working memory task that involved encoding and memoranda manipulation was not affected by time of day or relationship to sleep. Declarative memory is significantly improved by sleep in a sample of normal adolescents.

## Introduction

Several studies primarily in adults have shown that sleep improves procedural memory, i.e. skills and procedures [1], [2] as well as declarative memory [3]. REM and slow-wave sleep (SWS) have been implicated in memory consolidation [3]–[5]. Lack of REM sleep is associated with poor recall of visual location [6]. Decline in declarative memory consolidation is correlated with a decline in slow-wave sleep [7]. Spencer et al. observed similar initial procedural learning in older and younger adults; however, the older adults’ performance did not improve following sleep, suggesting that sleep dependent memory consolidation decreases with age [8]. This may reflect the disturbed sleep and disrupted SWS in the elderly [3], [8], [9]. Slow wave sleep increases until shortly before puberty and then shows a prominent drop across adolescence, decreasing by more than 60% between ages 10 and 20 years [10]. It is critical to understand the cognitive effects of normal sleep in order to understand the consequences of disrupted sleep. This is important since the majority of adolescents do not obtain the recommended amount of sleep and that disrupted sleep is a key symptom in most adolescent psychiatric and developmental disorders [11].

Backhaus et al. studied twenty-seven children with an average age of 10.1 years (range of nine to twelve), on a learned word pairs list, employing a within subject design and two post-learning assessments. They found that declarative memory was significantly increased immediately after an interval of sleep, as well as with delayed post-learning sleep [12]. As the authors had noted, no control task was administered to determine if circadian confounds were responsible for this increase in recall post sleep. Our study addressed this limitation by administering a control task and evaluating the effect of sleep on auditory declarative memory consolidation in early adolescence. Visual declarative memory has been reported to be enhanced following sleep in children; however, auditory declarative memory has not been previously studied [13].

## Methods

### Participants

Twenty female and twenty male adolescents, between the ages of 10 and 14, were recruited in a public middle school. The study was considered exempt by the institutional review board because it involved the use of educational tests without personal subject identifiers. In accord with the principals of the Declaration of Helsinki, subjects were asked to participate in a school class project and only told that they would be tested two times for about 15 minutes each time. Subjects with academic failure or accelerated academic performance or sleep problems were not included. The subjects agreeing to participate were grouped by sex and assigned to sleep or no sleep conditions with a separate randomization table for each group, to ensure a balanced design.

### Procedures

Subjects were tested in their homes in a quiet room without distractions for the duration of the learning and testing. The testing sessions were conducted during weekends or during school break. All subjects were given the paired-associate test, one of the standard tests of declarative memory [14], which consisted of repeating semantically related and unrelated pairs of words (e.g. tree/leaf; lamp/shoe), in a standardized manner. After each word pair was presented out loud, the subject repeated the pair out loud to ensure registration of the paired associate. The list of the same 10 pairs was administered three times in immediate succession. Subjects assigned to the sleep condition learned the paired associates at 9∶00pm (±30 minutes), and were tested for cued recall twelve hours later, after a night of sleep. The no-sleep group received the same paired-associate presentation at 9∶00am (±30 minutes) and was tested for recall twelve hours later, with no intervening sleep or naps. The control working memory task, letter-number, was given just prior to learning the paired-associate words and again just prior to being tested on the paired-associate words. The letter-number test was administered to control for possible circadian confounds and to control for attention and encoding. The letter-number control task (LN, immediate recall and reordering of letters and numbers) is a subtest of the WAIS-III (Wechsler Adult Intelligence Scale) and WMS-III (Wechsler memory Scale), the most widely used intelligence and memory scales. An increasing long series of mixed letters and numbers is read to the subject and the subject then orders the numbers and letters in ascending order, e.g. b3a1 is read and subject correctly responds with 13ab. The letters and numbers must be encoded and then manipulated to get the correct answer. Two versions of the letter-number task were used in random order. The number correct was scored for the paired-associate and the letter-number tests. The memory scores were transformed into Z scores to determine if outliers were present; an exclusionary Z score of ±2.57 was applied (1% of the normal distribution). Between group comparisons were calculated by students t-test (2 tailed) after testing for equal variances by Levene’s test, and ANCOVA as necessary. Within subject comparisons were calculated by paired t-test.

Subjects were instructed to eat their usual meals approximately one hour before learning the paired-associates and one hour before being tested on the paired-associates. Subjects were instructed to get a good night’s sleep. All the subjects included reported having had typical night of sleep and rated the quality of the sleep as good to very good prior to the testing.

## Results

The sleep group’s mean age was 12.9 compared to 12.4 for the non-sleep group (t = (1.52), df (1,38), p = 0.14). (See Table 1 for demographic characteristics and performance scores). There was no statistically significant sex difference in performance for either task.

**Table 1:** Demographic and performance scores for subjects in the sleep (n = 19) and no sleep (n = 18) conditions with outliers removed.

	Sleep	No Sleep	*P*
**Age**	12.95±1.05	12.4±2.15	0.14
**Female;Male**	10;9	8;10	
**Reported quality of sleep**	19 of 19 good	18 of 18 good	
**Letter-Number Correct Initial Test**	6.58±1.02	6.06±1.06	0.13
**Letter-Number Correct Second Test**	6.26±1.59 *	6.33±1.03 *	0.88
**Paired Associate Recall Correct After 12 Hours**	7.37±1.74	6.11±1.60	0.029

* not different from initial test.

Mean ± SD.

*p*’s are two-tailed tests.

**Figure 1:**
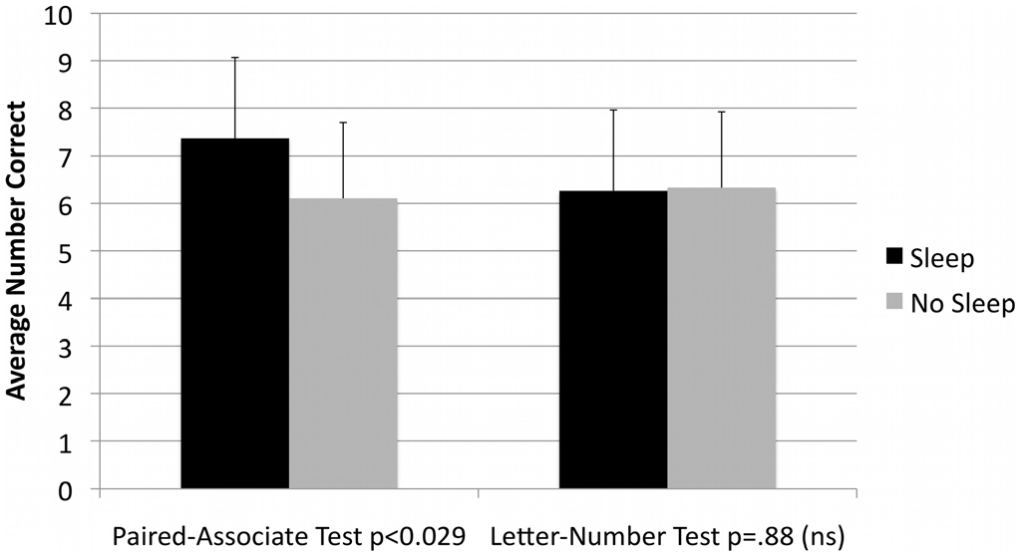
Average Number Correct in Sleep and No Sleep Categories. A histogram of mean number correct (± SD) for the Paired-Associate Test (PA) and Letter Number Test (Letter #), with (n = 19) (outliers removed) and without sleep (n = 18).

Three outliers were identified and removed; one high scoring subject assigned to the sleep and two lower scoring subjects assigned to no sleep. After removing outliers, 19 sleep subjects and 18 no- sleep subjects remained. The Levene’s Test showed equality of variances for all comparisons. The number correct on the letter-number control task at initial testing was 6.58 for the sleep group and 6.06 for the no-sleep group, (t = (1.54), df (1,35), p = 0.13). The letter-number correct score on the second administration was 6.26 and 6.33, respectively, (t = (-.16), df (1,35), p = 0.88), (Figure 1). There was also no statistically significant difference in performance for either group on letter-number task between the first and second administration (paired t test, p = 0.32 for sleep group and 0.45 for no-sleep group).

An increase of 20.6% in long-term memory (Figure 1) was found as measured by the number correct in the paired-associate test following sleep, compared to the group which was tested at the same time interval, but without sleep (p<0.029). When the three outliers are included, the number correct for recall of the paired-associates was statistically greater for the sleep group (7.5) compared to the no sleep group (5.9, t = (2.76), df (1,37), p<0.009), a 32.7% increase.

## Discussion

The paired-associate test is one of the standard tests of declarative memory and has been previously used to study declarative memory and the effects of sleep on declarative memory in adults and children [3]. All subjects were evaluated at the same two times of day, approximately 9 AM and 9 PM, using standardized conditions. Performance on the paired-associate test was significantly affected by sleep in our adolescent sample. In contrast, working memory performance as measured by the letter-number test, a standard subtest of the WAIS-III and WMS-III was not affected by time of day or in relation to sleep. Correct performance on the letter-number working memory task (LN) requires that the letters and numbers presented to the subject must be encoded and then correctly manipulated. We had 80% power to detect a standardized difference of.76 correct (∼11% change) or greater, following sleep. A small difference in working memory performance (<11%), however, may exist that could not be detected with our sample size.

The equal performance at both sessions and between groups on the LN supports the view that equal registration and encoding of the memoranda was comparable at both time points and between groups. Performance on the working memory control task did not change with the second session for either group, suggesting that the time of day had no effect on performance on the working memory control task. Consequently, the observed difference in paired-associate performance, i.e. consolidation of working memory, is most likely related to sleep itself and not any differences in encoding. Memory consolidation has been reported to be affected by sleep [1], [2], [8], [9]. Both REM and slow-wave sleep have been associated with improved memory [3]–[5]. Slow wave sleep particularly enhances declarative memory.^7^.

Our results are consistent with Gais et al.’s study of young males (mean age 17.4) showing that enhanced declarative memory was related to periods of sleep, and not to time of day effects [15]. Naps improve declarative memory regardless of time of nap [16] and closely resembled memory improvement after an eight-hour night of sleep [17]. In reviewing the timing of sleep and circadian rhythms, Diekelmann et al. conclude that sleep promotes memory consolidation independently of the time of day in which it occurs [3]. Voderholzer et al. studying 14–16 year old adolescents showed that several nights of sleep restriction did not impact memory consolidation nor performance in a working memory task, when two recovery night of sleep were provided, an effect they ascribed to a compensatory enhancement of SWS [18]. The paired-associate test begins as a working memory task and after a period of time with consolidation becomes a declarative memory task. Correct performance on the letter-number test and the paired-associate tests are dependent upon encoding the memoranda.

A limitation of this study is that we did not test for encoding strength by immediate recall after the administration of the paired-associate test. The letter-number test requires attention and encoding. An element of immediate recall is to prove that the subject was attending. This was assured by having the subjects read the words (similar to other learning tests like the CERAD and ADAS-COG) and supported by the finding of the performance on the letter-number test. It is likely that if immediate recall following each presentation was obtained, higher accuracy rates would have been observed. Recent studies have demonstrated that salience increases declarative memory performance [13], [19]. Nevertheless, our data demonstrate that sleep improves memory consolidation even in conditions where encoding has not been reinforced.

Neither time of day or sleep affected the performance on the letter-number test suggesting that the material was being learned and encoded. There is no evidence that memory consolidation depends on time of day independent of sleep. The lack of interference during sleep has been considered as a possible cause of the beneficial effects of sleep on declarative memory, i.e. there are no daytime demands to interfere with memory consolidation. Our design tested subjects on non-school days, thus mitigating the effects of interference of memory consolidation during the day by learning competition and other demands of a normal school day. Gais et al. controlled for waking associated interference and found no effect of interference on memory [15]. In a review of controversy regarding whether absence of interference accounts for memory improvement during sleep, Ellenbogen at al. point out “although sleep might passively protect declarative memories from interference, consolidation must also occur during sleep for the memories to become resistant to interference the following day”. Based on their review of related animal and human studies, they point out that “hippocampus-dependent memories are reactivated during sleep, and that this reactivation leads to strengthened memory traces”, finally concluding “that specific, sleep-dependent, neurobiological processes directly lead to the consolidation of declarative memories” [1]. Diekelmann et al. hypothesized that both encoding and sleep-dependent consolidation during sleep involve prefrontal-hippocampal circuitry [3].

Children have high amounts of slow wave sleep and sleep in general. Sleep has been shown to improve declarative and procedural memory in children and older age groups. Subjects were asked about their sleep and confirmed that they had a typical night sleep, consisting of 8–10 hours of sleep, average for adolescents [20]. We did not, however, specifically measure sleep. Lack of sleep can result in poor cognitive performance, which was not observed in our sample, and is consistent with the subjects’ report of a good night sleep and that poor sleepers were excluded from the sample.

A cross-over design would have provided additional confirmation at the individual subject level in contrast to our parallel group design. Our study was limited as the sample was opportune, from a California middle school, and was not epidemiologically based. No subjects approached declined to participate. No accelerated or failing students were included, although this was not a strict exclusion criterion. There were 3% African-American, 5% Asian, and 92% Caucasian. The sample population reflected the general school population in this geographic area, although Asians were underrepresented (12.8%).

Our sample size was relatively small and limited to early adolescence, ages 10–14, although twice the sample of Prehn-Kristensen et al. who found 10 to 13 year olds improved visual memory following sleep, especially to emotional pictures [13]. The 10–14 age group was deliberately chosen because of the importance of declarative memory on adolescent school performance and related social functioning [21]. Marked changes in sleep and sleep architecture are a defining feature of adolescence [22]. Disorders of adolescence frequently disrupt sleep. Twenty-five to forty percent of adolescents have sleep disorders that can have an important effect on daytime school and consequent social functioning [23]. Sleep disorders are even more prevalent in adolescents with psychiatric disorders and developmental disabilities [24]. It is important to have data on the effects of normal sleep on declarative memory in normal adolescents to better understand the consequences of lack of sleep and abnormal sleep patterns.

Given the importance of adolescent memory on school performance and consequent social functioning, a fuller understanding of the effects of sleep on memory consolidation is needed. Other studies are needed to investigate the specific effects of sleep on other types of memory, such as visual, procedural, and emotional. Understanding the role of normal sleep on memory consolidation in adolescence is critical in identifying the consequences of disrupted sleep in adolescent disorders and their treatment.
